# *In Vitro* Biocompatibility and Endothelial Permeability of Branched Polyglycidols Generated by Ring-Opening Polymerization of Glycidol with B(C_6_F_5_)_3_ under Dry and Wet Conditions

**DOI:** 10.1021/acs.biomac.4c00210

**Published:** 2024-05-04

**Authors:** Carlo Andrea Pagnacco, Marcelo H. Kravicz, Francesco Saverio Sica, Veronica Fontanini, Estibaliz González de San Román, Reidar Lund, Francesca Re, Fabienne Barroso-Bujans

**Affiliations:** † Donostia International Physics Center (DIPC), Paseo Manuel Lardizábal 4, Donostia−San Sebastián, 20018, Spain; ‡ Centro de Física de Materiales, CSIC-UPV/EHU, Paseo Manuel Lardizábal 5, Donostia−San Sebastián, 20018, Spain; § School of Medicine and Surgery, University of Milano-Bicocca, Milano, 20854, Italy; ∥ Department of Life Sciences, University of Trieste, Trieste, 34127, Italy; ⊥ POLYMAT, Joxe Mari Korta Center, University of the Basque Country UPV/EHU, Avda. Tolosa 72, Donostia−San Sebastián, 20018, Spain; # Department of Chemistry, University of Oslo, Postboks 1033, Blindern, Oslo, 0315, Norway; ∇ Hylleraas Centre for Quantum Molecular Sciences, University of Oslo, Postboks 1033, Blindern, Oslo, 0315, Norway; ○ IKERBASQUE - Basque Foundation for Science, Plaza Euskadi 5, Bilbao, 48009, Spain

## Abstract

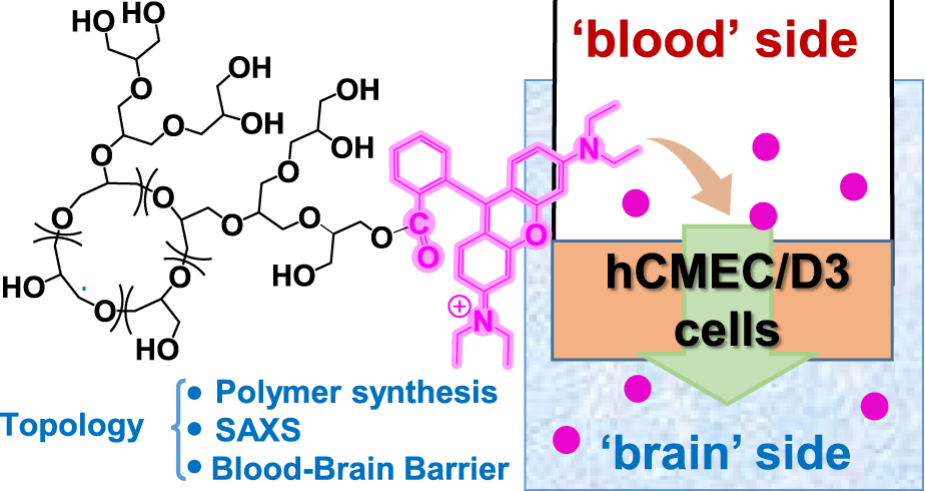

Polyglycidol or polyglycerol (PG), a polyether widely used in biomedical applications, has not been extensively studied in its branched cyclic form (*bc*PG), despite extensive research on hyperbranched PG (HPG). This study explores the biomedical promise of *bc*PG, particularly its ability to cross the blood–brain barrier (BBB). We evaluate *in vitro* biocompatibility, endothelial permeability, and formation of branched linear PG (*bl*PG) as topological impurities in the presence of water. Small angle X-ray scattering in solution revealed a fractal dimension of approximately two for *bc*PG and the mixture *bc+bl*PG, suggesting random branching. Comparisons of cytotoxicity and endothelial permeability between *bc*PG, *bc+bl*PG, and HPG in a BBB model using hCMEC/D3 cells showed different biocompatibility profiles and higher endothelial permeability for HPG. *bc*PG showed a tendency to accumulate around cell nuclei, in contrast to the behavior of HPG. This study contributes to the understanding of the influence of polymer topology on biological behavior.

## Introduction

Polyglycidol, also known as polyglycerol (PG), is a polyether frequently used in biomedical and pharmaceutical applications. Its popularity stems from its biocompatibility and notable functionality, enabling the binding of molecules with biomedical significance.^1−3^ Various PG architectures have been studied including linear, dendritic, and hyperbranched structures. Hyperbranched PG (HPG) is characterized by a random distribution of hydroxyl groups throughout its globular structure, which in contrast to perfect dendrimeric structures the hydroxyl groups are not located at the same distance from the core. HPG is typically obtained through the ring-opening multibranching polymerization (ROMBP) of glycidol using partially deprotonated multifunctional hydroxyl initiators.^4^ HPG has demonstrated exceptional encapsulation capabilities for both hydrophobic and hydrophilic molecules, making it highly valuable for controlled and targeted drug delivery, improving therapeutic efficacy while reducing side effects.^5,6^ HPG has been investigated for use in 2D surface^3,7,8^ and nanoparticle (NP) coatings,^8−11^ providing antifouling properties that reduce the adhesion of proteins, cells, fungi, and bacteria.^12^

The influence of the architecture of PGs on their biological properties and the unique advantages of PGs over the widely used PEG have also been addressed in a number of studies, as reviewed elsewhere.^1−3^ For example, a comparative blood compatibility study of linear PG (LPG), HPG, and PEG with molecular weights of ∼100 kDa showed that unlike PEG, LPG and HPG did not induce undesirable red blood cell aggregation and hemolysis.^13^ In that study, LPG showed shorter blood circulation, higher renal clearance, and deformability compared to HPG. Other i*n vitro* and *in vivo* biocompatibility studies of LPG and HPG with lower molecular weights of ∼6 kDa showed that the topology had no effect on their biocompatibility.^14^ The influence of the architecture of the PGs on their biological activity was also evaluated in bioconjugation studies. Comparative studies of LPG-, HPG- and PEG-anakinra bioconjugates showed that LPG conjugates exhibited significantly better proteolytic stability than their PEG analogues, and all conjugates significantly prolonged the elimination half-life of anakinra, with LPG and PEG conjugates prolonging it 4-fold compared to the unmodified protein.^15^ Other bioconjugation studies were performed to compare the performance of LPG- and PEG-modified proteins (*e.g.*, interleukin-4 WT,^16^ interferon α2a,^17^ and erythropoietin^18^) in terms of their bioactivity. The PEGylated and PGylated forms of interleukin-4 WT and erythropoietin showed similar biological activities, whereas those of interferon α2a showed different pharmacokinetics. In this case, a faster initial distribution rate was observed for the LPGylated-interferon α2a bioconjugates compared to the PEGylated ones.^17^ HPG has shown significantly improved properties compared to PEG when used to coat NPs.^8−10^ Some of these properties include longer blood circulation and significantly lower liver accumulation for HPG-based NPs compared to analogous PEG-based NPs, as well as higher stability in suspension and better therapeutic efficacy against tumors *in vivo*.^9^

HPGs also exhibit interesting physical properties related to their globular structure, which have been extensively studied over the past 25 years.^1−3^ For instance, high-molar-mass HPGs exhibit low intrinsic viscosity (*e.g.*, 6.15 mL/g for an HPG structure with *M*_w_ of 9.3 × 10^6^ Da), which is similar to globular proteins in aqueous solutions, and which is substantially higher than that of an equivalent PEG of *M*_w_ of 11 × 10^6^ Da (2600 mL/g).^19^ The intrinsic viscosity for HPGs at moderate and high *M*_w_ has been observed to be independent of the molecular masses, in contrast to PEG, whose intrinsic viscosity follows a Mark–Houwink–Sakurada behavior with an exponent of ∼0.7.^19^ HPGs are soluble in water at high concentrations (>400 mg/mL), in contrast to PEG, which tends to aggregate, exhibiting both upper and lower critical solution temperature phenomena in water.^20^

**Scheme 1:**
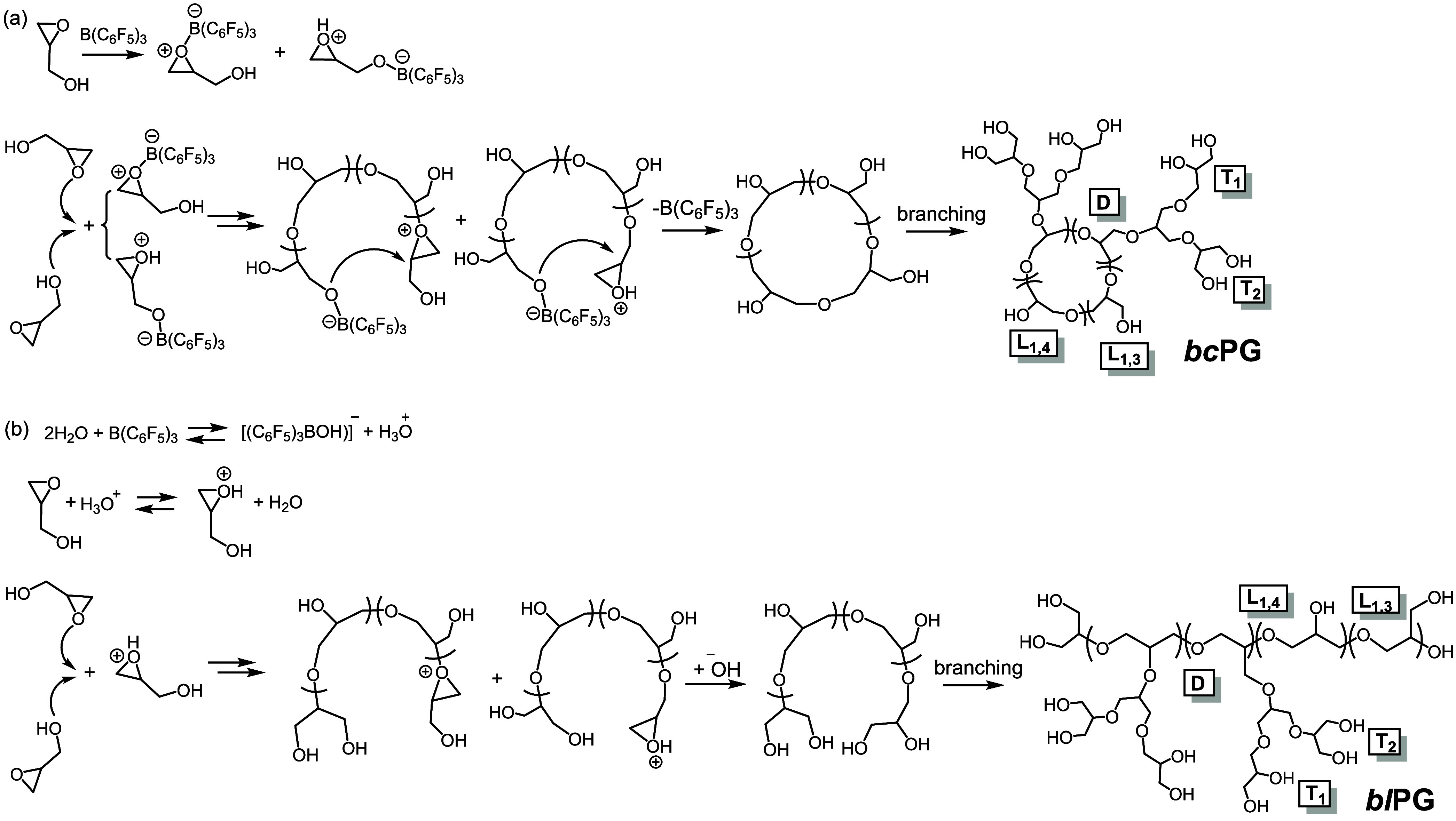
Competitive Mechanisms of Initiation of Gly by (a) B(C_6_F_5_)_3_ and (b) H_3_O^+^ Leading to the Formation of Cyclic-Core and Linear-Core Branched Structures, Respectively^26^ Both topologies are formed by linear (L_1,3_ and L_1,4_), dendritic (D), and terminal units (T_1_ and T_2_).

Branched cyclic-core PG (*bc*PG) has been obtained by zwitterionic ring-opening polymerization (ZROP) of glycidol (Gly) with tris(pentafluorophenyl)borane [B(C_6_F_5_)_3_],^21,22^ a mechanism also called electrophilic zwitterionic ring expansion polymerization (eZREP)^23,24^ (Scheme 1a). Initiation occurs by reaction of B(C_6_F_5_)_3_ with both the epoxide and alcohol oxygen of Gly. The generated oxonium ions are prone to attack by further epoxide and hydroxyl groups of Gly at the methine (alfa) and methylene (beta) carbons, leading to a chain structure formed by L_1,3_ and L_1,4_ linear units. The active chain end (ACE) ROP mechanism is the one operating *via* the attack of epoxide ring to oxonium ions located at the chain end, and the activated monomer (AM) ROP mechanism is the one occurring *via* the attack of hydroxyl groups.^25^ Both charges of the propagating zwitterionic chain travel together, facilitating the incorporation of a new Gly monomer into the structure while maintaining the preformed cyclic structure. Termination occurs by end-biting. Additionally, hydroxyl side groups can react with the growing chains to form branches in dendritic (D) structures and T_1_ and T_2_ terminal units.^21^

In this polymerization mechanism, water may play a role in both the termination and initiation processes. A chain transfer reaction with water would result in the formation of chains terminated with hydroxyl groups, thereby inhibiting the formation of cyclic structures. Water can also initiate the polymerization by protonating Gly epoxide through the hydronium ions formed in a reaction between B(C_6_F_5_)_3_ and two water molecules (Scheme 1b).^26^ This mechanism competes with that producing *bc*PG by forming linear-core branched PGs (*bl*PG).^26^ Of particular significance is that both structures, *bc*PG and *bl*PG, are formed from the beginning of the polymerization reaction due to similar kinetics.^26^ In practical applications, the existence of *bl*PG chains in *bc*PG samples might be undesirable as they could be considered topological impurities. Therefore, further studies of the formation of *bl*PG structures and their properties are warranted.

The exploration of *bc*PG structures is presently ongoing for diverse applications in material synthesis. These applications include adhesives,^27^ stabilizers of pigment particles,^28^ and self-healing materials.^29^ The distinctive topology of *bc*PGs played a crucial role in influencing the performance and properties of these materials. In the biomedical field, we also expect *bc*PG to demonstrate favorable performance comparable to well-established HPGs and HPG-based materials.^1−3,30−32^ Their hyperbranched structure, similar to HPGs, may impart desirable properties such as enhanced biocompatibility, low toxicity, and versatility in functionalization. A deeper understanding of the interactions between *bc*PGs (and its topological impurity *bl*PG) and biological systems is needed.

The objective of this study was 2-fold. First, we aimed to investigate the impact of water on the formation of *bl*PG structures in *bc*PG samples generated by ZROP. Second, our goal was to assess the biocompatibility characteristics of these PG structures and their ability to cross a model of biological barriers. Among biological barriers, the most complex and challenging to overcome is the blood–brain barrier (BBB). The BBB is a highly selective and protective physiological barrier composed of specialized endothelial cells that tightly regulate the passage of substances from the bloodstream into the brain, maintaining the unique microenvironment required for proper neural function.^33^ By addressing these dual objectives, we aimed to provide a comprehensive understanding of the factors influencing the biological response of PG structures from a topological perspective.

To achieve these aims, the polymerization of Gly was conducted under bulk conditions using B(C_6_F_5_)_3_ as a catalyst and varying amounts of water, ranging from dry conditions to an extremely large excess of water ([H_2_O]_0_/[B(C_6_F_5_)_3_]_0_**=** 600/1). Matrix-assisted laser desorption/ionization time-of-flight mass spectrometry (MALDI-ToF MS) revealed an increase in *bl*PG structures with increasing water content in the system, alongside *bc*PG topologies that are generated under all conditions. These results indicate that *bc*PG is the most favored thermodynamic product, while obtaining *bl*PG with a topological purity is not feasible. Small angle X-ray scattering (SAXS) data were obtained to analyze the structure and chain conformation of the synthesized *bc*PGs, revealing more compact structures (smaller *R*_g_ values) in the absence of water, where *bl*PGs do not form, compared to the polymerization performed in the presence of water. Then, PGs were labeled with approximately 3 mol % of the fluorescence dye rhodamine B to facilitate tracking in biological assays. These experiments showed that PGs can be taken up by the cells and that polymer concentrations up to 100 μg mL^–1^ are well tolerated. By using HPG as a control topology, the results indicated that the topology of the PGs plays an essential role in the BBB crossing *in vitro*.

## Experimental Section

### Materials for Polymer Synthesis and Functionalization

Glycidol (Gly) and tetrahydrofuran (THF) were distilled from CaH_2_ under reduced pressure, and B(C_6_F_5_)_3_ was sublimated. Rhodamine B (RhB), sodium hydride (NaH), dichloromethane, methanol, and diethyl ether were purchased from Sigma-Aldrich and used as received. 4-Dimethylaminopyridine (DMAP) and *N*-(3-(dimethylamino)propyl)-*N*′-ethylcarbodiimide hydrochloride (EDC) were purchased from Acros Organics. *N,N*-dimethylformamide (DMF) and 1,1,1-trimethylolpropane (TMP) were purchased from Thermo Scientific.

### Synthesis of *bc*PG

In a typical reaction (Entry 7, Table 1), 3 mL of Gly (0.045 mol) was cooled to 0 °C in a round-bottom flask under propeller stirring. 29 mg of B(C_6_F_5_)_3_ (57 μmol) was dissolved in 0.5 mL of CH_2_Cl_2_ and gradually added to Gly. The reaction was stirred for 24 h. Termination was performed by adding methanol. ^1^H NMR of crude reactions was recorded in D_2_O to determine the monomer conversion. The reaction was purified by precipitation in acetone from a methanol solution and centrifugation. Finally, samples were dried at 120 °C in a vacuum oven overnight (yield = 90 wt %).

**Table 1:** Polymerization of Gly in “Dry” and “Wet” Conditions

entry	[Gly]_0_/[H_2_O]_0_/[B(C_6_F_5_)_3_]_0_	*T* (°C)	*t* (h)	*M*_n_^a^(theo)(kg/mol)	*M*_n_^b^(GPC)(kg/mol)	*M*_p_^b^(GPC)(kg/mol)	Đ^b^	Conversion (mol %)
1	594/0/1	0	24	44.0	9.7	12.8	2.3	100
2	594/15/1	0	48	2.9	3.5	5.8	2.1	98
3	594/29/1	0	48	1.4	1.9	2.4	2.0	94
4	594/58/1	0	48	0.7	1.9	2.8	2.6	99
5	594/292/1	40	24	0.2	1.6	1.8	1.2	95
6	594/292/1	60^c^	24	0.2	0.6	0.9	1.9	99
7	800/0/1	0	24	59.2	9.7	10.3	2.0	100
8	800/29/1	0	24	2.0	3.8	5.1	1.7	99
9	800/600/1	60^c^	24	0.1	0.8	0.9	1.2	95
10	800/1200/1	60	24	0.03	0.3	0.5	1.6	90

a for “wet” polymerizations and  for “dry” polymerizations.

b *M*_n_ and *M*_p_ were obtained by using GPC-RI-MALS in DMF + 0.1% LiBr (*dn*/*dc* = 0.054 mL/g).^38^

c The viscosity increased rapidly within a few minutes of initiating the polymerization.

### Synthesis of *bc*+*bl*PG

In a typical reaction (Entry 8, Table 1), 3 mL of Gly (0.045 mol) was cooled to 0 °C in a round-bottom flask under propeller stirring. 29 mg of B(C_6_F_5_)_3_ (57 μmol) was dissolved in 0.5 mL of CH_2_Cl_2_, mixed with 30 μL of water, and gradually added to Gly. Termination was performed by adding methanol. ^1^H NMR of crude reactions was recorded in D_2_O to determine the monomer conversion. The reaction was purified by precipitation in acetone from methanol solution and centrifugation. Finally, samples were dried at 120 °C in a vacuum oven overnight (yield = 50 wt %).

### Synthesis of HPG

HPG was synthesized following a literature procedure.^34^ Briefly, TMP (18.8 mg, 0.140 mmol) and NaH (1.0 mg, 0.042 mmol) were added into a flame-dried round-bottomed flask in a glovebox. Then, 1 mL of dry THF was added, and the mixture was stirred at 95 °C for 30 min. The activation of the hydroxyl group was proved by a pale-yellow coloration of the suspension. Then, Gly (1.1 g, 14.8 mmol) was added using a syringe pump during 24 h under an argon atmosphere. After the reaction completion, the polymer was solubilized in a few mL of HCl 0.1 N in MeOH and poured into 500 mL of diethyl ether. Then, the polymer was collected by centrifugation (monomer conversion = 96%, *M*_n_ = 5 kDa, PDI = 1.6, degree of branching = 0.50).

### Fractionation of Polymers

Fractions of Entry 7 were obtained by fractional precipitation from a methanol solution (13 mL) of 1.5 g of polymer. Initially, 6 mL of diethyl ether was added, causing the precipitation of the first fraction, which was separated by centrifugation. The remaining solution was successively precipitated with further additions of about 7 to 15 mL of diethyl ether. Similarly, fractions of Entry 8 were produced from a methanol solution (10 mL) of 0.8 g of the polymer. Initial amount of 7.5 mL of diethyl ether was needed to generate the first polymer fraction. Next fractions were generated by successively adding 1.5 to 4 mL of diethyl ether.

### Functionalization of Polyglycidol with Rhodamine B

50 mg of PG (HPG, Entry 7/F3 and Entry 8/F1) (0.68 mmol of OH groups) was dissolved in 5 mL of anhydrous DMF. 12 mg of DMAP (0.10 mmol), 60 mg of EDC (0.38 mmol), and 159 mg of RhB (0.33 mmol) were added. The mixture was stirred at room temperature overnight. The reaction was directly poured into 500 mL of cold diethyl ether, and the polymer was separated by centrifugation and dialyzed in water. The product was finally dried by lyophilization. The degree of functionalization was determined by integration of the CH_3_ signal of RhB (, 3*H*) at 1.3 ppm and PG signals (*I*_PG_, 5*H*) at 3.4–4.1 ppm in the ^1^H NMR spectra. In the calculation, the corresponding integral of RhB CH_2_ signal (, 2*H*) at 3.7 ppm () was subtracted from the signal at 3.4–4.1 ppm. RhB-functionalized *bc*PG and *bc+blPG* were verified by ^19^F NMR to be free of B(C_6_F_5_)_3_.

### Cell Line

Immortalized human cerebral microvascular endothelial cells (hCMEC/D3) were provided by Dr. S. Bourdoulous (Institut Cochin, Inserm, Paris, France) and used as a model of the brain capillary endothelium.^35^ Cells at passage between 25 and 35 were seeded on tissue culture flasks and pretreated with rat tail collagen type I (0.05 mg/mL). Cells were grown in complete culture medium (EBM-2 supplemented with 10% FBS, 1% chemically defined lipid concentrate (CDLC), 1% penicillin/streptomycin (P/S), 10 mM Hepes, 5 μg/mL ascorbic acid, 1 ng/mL bFGF, and 1.4 μM hydrocortisone) and maintained at 37 °C, 5% CO_2_. The culture medium was changed every 2 days.

### *In Vitro* BBB Model

The *in vitro* BBB model was prepared and characterized, as previously described,^36^ using hCMEC/D3 cells. Briefly, cells were seeded (56,000 cells/cm2) onto collagen-coated (150 μg/mL rat tail collagen type 1; Gibco, Thermo Fisher Scientific) transwell filters (polyester 12-well, pore size 0.4 μm, translucent membrane inserts 1.12 cm^2^; Costar) to establish a polarized monolayer. The cell monolayer separates into two compartments: an apical one (0.5 mL) representing the blood and a basolateral one (1 mL) representing the brain. Cells were grown for 3 days in a complete EBM-2 medium. After 3 days, the medium was replaced with EBM-2 supplemented with 5% FBS, 1% CDLC, 1% P/S, 10 mM Hepes, 5 μg/mL of ascorbic acid, 1.4 μM hydrocortisone, and 10 mM LiCl. The formation of junctions was evaluated by measuring TEER, monitored with STX2 electrode Epithelial Volt-Ohm meter (World Precision Instruments, Sarasota, FL, United States) and the paracellular permeability to TRITC-dextran 4400 Da (λecc = 557 nm, λem = 572 nm) (Sigma-Aldrich, Milano, Italy).

### Cell Viability Assay

The effect of chain topology on hCMEC/D3 viability was assessed by the MTT assay using RhB-functionalized PG samples (*bc*PG, *bc+bl*PG, and HPG).^36^ hCMEC/D3 cells were seeded in a 96-well plate at a density of 20,000 cells per well. Different doses of PG, ranging from 1 to 1000 μg mL^–1^, were added to the culture medium. After 24 h, the assay was performed as per the manufacturer’s protocol, and absorbance was measured at 690 and 570 nm using a microplate reader (SPECTROstar Nano, BMG LABTECH, Ortenberg, Germany). Results are presented as the mean of three independent experiments ± SD considering untreated cells as 100% of the cell viability. Data were analyzed using GraphPad Prism 8 software, and the statistical analysis was performed by two-way ANOVA.

### Characterization Techniques

Monomer conversion was determined by^1^H NMR. The spectra of polymer samples synthesized in flasks were acquired at 25 °C on a Bruker Avance Neo 400. Around 5 mg of crude polymer samples was dissolved in approximately 0.5 mL of D_2_O. Signal integration of free monomer at 2.6–2.9 ppm (CH_2_OH, 2H) and that combining PG (5H) and free monomer (CH_2_OCH, 3H) at 3.1–4.0 ppm were used in the calculation. Relative abundance of structural units and degree of branching (DB) was determined by inverse gated ^13^C NMR following previous works.^21,22^ The spectra were acquired on a Bruker Avance Neo 500. Around 50 mg of purified polymers was dissolved in 0.5 mL of D_2_O. DB was calculated from DB = 2D/(2D + L), where, D and L = L_1,3_ + L_1,4_ are the relative abundance of dendritic and linear structures, respectively.^4^ Note that the eq DB = (D + T)/(D + T + L), where *T* = T_1_ + T_2_ is the relative abundance of terminal units, would lead to an overestimation of DB for small or low branched molecules, as explained in ref^37^ and shown in Table S1.

GPC data were acquired using a Nexera instrument from Shimadzu using a refractive index detector (RID-20A, Shimadzu) and MALS detector (λ = 663.89 nm, miniDawn, Wyatt). DMF containing 0.1% LiBr with a flow of 1.0 mL/min was used as an eluent. Separation was performed at 50 °C by using a CTO 40C column oven and Polargel-M Guard 50 × 7.5 mm and Polargel-M 300 × 7.5 mm, 8 μm, GPC columns. The absolute molecular weights were determined using a d*n*/d*c* value^38^ of 0.054 mL/g and Astra 8.1 software from Wyatt Technology.

MALDI-TOF MS measurements were performed on a Bruker Autoflex Speed system (Bruker, Germany) equipped with a Smartbeam-II laser (Nd:YAG, 355 nm, 2 kHz). Spectra were acquired in reflectron mode; each mass spectrum was the average of 10,000 shots. The laser power was adjusted during the experiments. Polymer samples were dissolved in MeOH at a concentration of 10 mg/mL. Alpha-cyano-4-hydroxycinnamic acid (CHCA, Sigma-Aldrich) was used as a matrix. The matrix was dissolved in MeOH at a concentration of 20 mg/mL. Lithium trifluoroacetate (LiTFA, Sigma-Aldrich) was used as a cation donor (10 mg/mL dissolved in MeOH). The polymer samples were mixed with the matrix and salt in a 10:10:1 (matrix/polymer/salt) ratio. Approximately 0.5 μL of the obtained solution was spotted by hand on the ground steel target plate and allowed to dry in the air. Spectra were accumulated and processed using FlexControl (v3.4) and FlexAnalysis software (v3.4), respectively. Peaks were detected in SNAP mode with a signal-to-noise threshold of 3.00 before being processed with a Savitzky–Golay smoothing algorithm (0.05 *m*/*z* width, one cycle) and “TopHat” baseline subtraction. External calibration was performed in quadratic mode with a mixture of different polystyrene standards (PS, Varian).

Differential scanning calorimetry (DSC) measurements were carried out on ∼5 mg specimens using a Q2000 TA Instruments. All samples were measured by placing them in aluminum pans without using lids, following confirmation that the type of pan significantly influenced data reproducibility.^21^ The sample was first cooled from room temperature to −100 °C and then heated to 150 °C at 10 °C/min (first heating run). Then, samples were cooled back to −100 °C at 10 °C/min and finally heated to 150 °C at 10 °C/min (second heating run). A helium flow rate of 25 mL/min was used throughout. The glass transition temperatures (*T*_g_) were determined from the maximum of the first derivative in the second heating run.

The SAXS experiments were performed using the automated BM29 bioSAXS beamline at the ESRF, Grenoble, France. For technical details, we refer to the ref^39^. The data were obtained using an energy of 12.5 keV and detector distance 2.87 m covering a Q-range (Q = 4π sin (θ/2)/λ, λ is the wavelength, θ is the scattering angle) of about 0.0047 Å-1< Q < 0.5 Å^–1^. The data were calibrated to an absolute intensity scale using water as a primary standard.

### Data Modeling

The total intensity can be written as
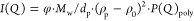
1

φ is the volume fraction of the polymer, *M*_w_ is the molecular weight, *d*_p_ is the solution density of the polymer, and ρ_p_ and ρ_0_ are the scattering length densities of the polymer and buffer, respectively. The solution density required to estimate ρ_p_, was measured using an Anton Paar DMA5000 densitometer and gave *d*_p_ = 1.34 g/mL for polyglycidol (Entry 7 sample). The scattering length density is calculated according to
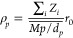
2where *M*_p_ is the molecular weight of the monomer and *r*_0_ is Thomson radius.

For the form factor, *P*(*Q*)_poly_, either a random chain form factor described by the Debye function for Gaussian chains, P(*Q*)_Debye_ or a general form factor for arbitrary chain statistics, P(*Q*)_Beaucage_,^40^ described by
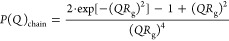
3where *R*_g_ is the radius gyration of the polymer chain.
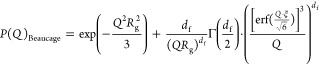
4where *R*_g_ is the radius of gyration, *d*_f_ is the fractal dimension (*d*_f_ = 2 and 1.7 for theta and good solvent respectively). Γ(x) is the gamma function, and erf(x) is the error function.

For the density measurements of the different surfactants, a DMA 4500 M liquid/solution density meter from Anton Paar was used. The specific volume of the polymer was calculated according to  where *w*_p_ is the weight fraction, *d*_0_ is the solvent (water) density and *d*_sol_ is the polymer solution density.

### Confocal Laser Scanning Microscopy

CLSM was employed to estimate the uptake and the intracellular localization of the fluorescently labeled PGs. CLSM pictures were taken using an LSM710-inverted confocal laser scanning microscope equipped with a Plan-Neofluar 63×/1.4 oil objective (Carl Zeiss, Oberkochen, Germany). Excitation was performed using two ultraviolet–visible-laser diodes 25 mV (405–488) and Ar-laser (540 nm) at 10% intensity. The pinhole was set to 1A. Image acquisition was performed sequentially to minimize cross-talk between the fluorophores. The hCMEC/D3 cells were incubated at 37 °C with PG for 3 h, rinsed three times with PBS, and fixed with a 10% (v/v) formalin solution. After three washes with PBS, cells were permeabilized with 0.2% (v/v) Triton X-100 in PBS for 15 min, then rinsed twice and incubated with a solution of 1% (v/v) phalloidin (actin filaments staining) in PBS for 1 h, then with 20 μM DAPI (nuclear staining) in PBS for 10 min. After three washes, the samples were mounted using poly(vinyl alcohol) mounting medium (Sigma-Aldrich). All washes were performed with PBS.

## Results and Discussion

### Synthesis of Polyglycidol

**Figure 1:**
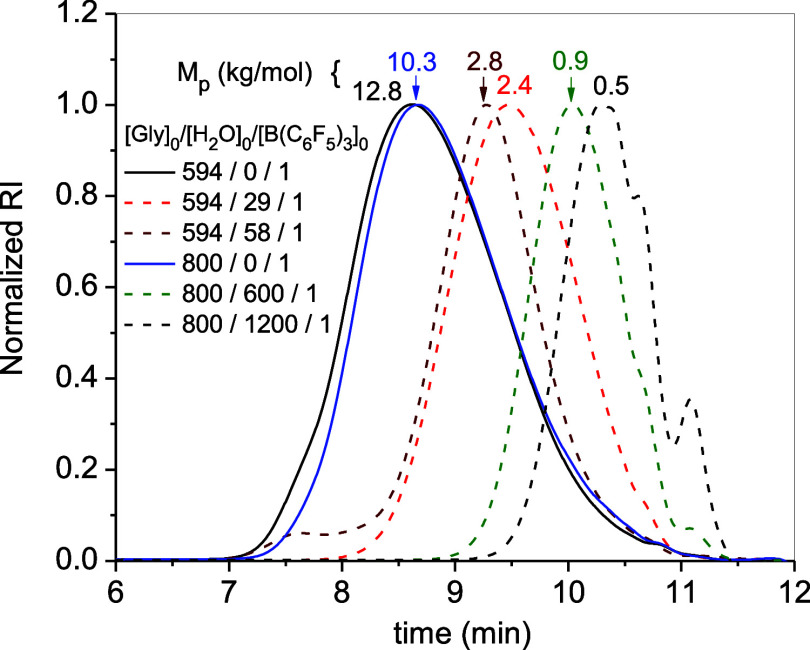
GPC data (DMF + 0.1% LiBr) of polyglycidols synthesized in “dry” and “wet” conditions. *M*_p_ values obtained by MALS-RI (d*n*/d*c* = 0.054 mL/g).^38^

*bc*PG was synthesized by reaction of Gly with B(C_6_F_5_)_3_ in the absence of water (hereafter “dry” polymerization) under bulk conditions at 0 °C for 24 h by using [Gly]_0/_[B(C_6_F_5_)_3_]_0_ = 594 and 800 (Entries 1 and 7, Table 1). The effect of water on the PG structure was investigated with a series of reactions carried out in the presence of varying amounts of water (hereafter “wet” conditions) and by changing the temperature (Entries 2–6 and 8–10, Table 1). The results showed that the *M*_n_ obtained experimentally was higher than those calculated theoretically in all cases of “wet” polymerization considering the initial molar concentration ratio of [Gly]_0/_[H_2_O]_0_, and that it decreased by increasing the amount of water. The latter observation was expected from an increase in the number of transfer reactions with free water. In the absence of water, the theoretical *M*_n_ calculated considering B(C_6_F_5_)_3_ as the initiator does not conform to [Gly]_0/_[B(C_6_F_5_)_3_]_0_ due to the release of the catalyst upon cyclization with the consequent reinitiation of the monomer, as previously described in ZROP with B(C_6_F_5_)_3_.^21,41^ As a result, the *M*_n_ obtained experimentally under “dry” conditions is, in all cases, smaller than the theoretical ones. The dispersity values of synthesized polymers were nearly 2 and decreased to values below 2 at very high water content. GPC data of representative samples synthesized in “dry” and “wet” conditions are compared in Figure 1. The molecular weight of the peak maxima (*M*_p_) accounts for the peak shift toward lower values with increasing amounts of water. At very large amounts of water, [H_2_O]_0_/[B(C_6_F_5_)_3_]_0_ > 250, the polymerizations became too slow. To alleviate this problem, we investigated raising the temperature to 60 °C, but in some cases, it resulted in fast reactions manifested in a sudden increase in viscosity and the release of white smoke (Entries 6 and 9). This response is a sign of an autoacceleration caused by a fast initiation.

The analysis of the relative abundance of structural units and the degree of branching (DB) is shown in Table 2 (NMR spectra in Figures S1–S3). In going from Entry 1 to Entry 5, and from Entry 7 to 10, the amounts of T_1_ units increase with a concomitant decrease of D units (and DB), a fact that can be directly related to an increasing number of transfer reactions with water and the consumption of active species. The less likely T_2_ structure (5–6%) seems to be independent of the amount of water in the reaction. Concerning the amounts of linear structures, L_1,3_ and L_1,4_, they are formed with equal probability in the samples, except when the reaction is violent at 60 °C, where the amounts of L_1,3_ exceed those of L_1,4_ (Entries 6 and 9).

**Table 2:** Relative Abundance of Structural Units and Degree of Branching (DB) of the PG Samples

	relative abundance (%)						
entry	*D*	L_1,3_	L_1,4_	T_1_	T_2_	L_1,3_/ L_1,4_	DB
1	23	27	27	18	5	1.0	0.46
2	24	29	29	13	5	1.0	0.45
3	20	25	28	22	5	0.9	0.43
4	16	25	27	27	5	0.9	0.38
5	11	27	25	32	5	1.1	0.30
6	18	36	20	20	6	1.8	0.39
7	23	27	28	17	5	0.9	0.46
8	21	29	26	19	5	1.1	0.43
9	10	32	21	31	6	1.5	0.27
10	6	25	22	42	5	1.1	0.20

**Figure 2:**
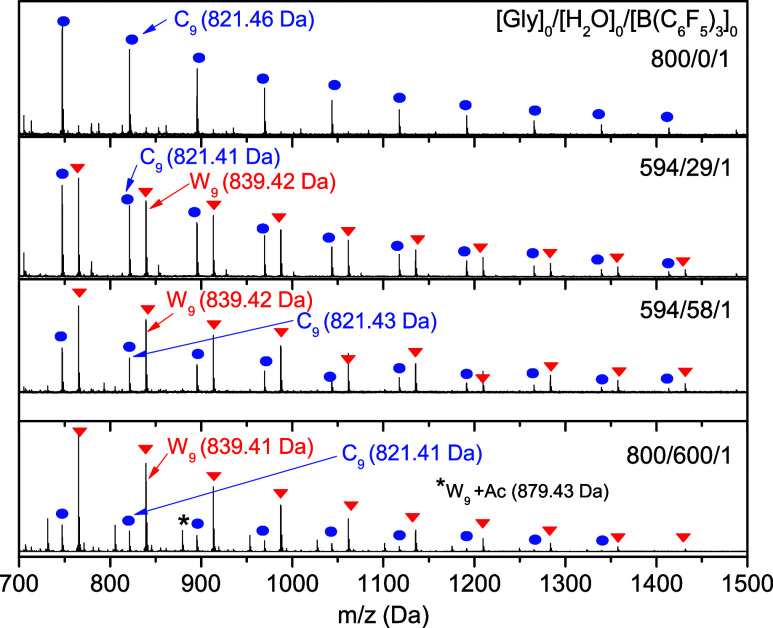
MALDI-ToF MS (reflectron mode) data of PGs synthesized in the absence and presence of water (Entries 3, 4, 7 and 9, Table 1). Li^+^ was used as a cation ionization agent. *W*_n_ + *A*c refers to *W*_n_ species that reacted with acetone upon precipitation and formed acetal species (W_n_ + 40 Da).

MALDI-ToF-MS data of representative samples synthesized in “wet” conditions exhibited high-intensity peaks with *M*_obs_ (*W*_n_) = n*M*_Gly_ +  + *M*_Li_, accompanied by peaks of lower intensities with *M*_obs_ (*C*_n_) = n*M*_Gly_ + *M*_Li_ of lithium-complexed chains (Figure 2). *C*_n_ indicates the formation of cyclic chains. The most likely structure is that in which a cyclic core is surrounded by branches (referred above as *bc*PG).^21,26^*W*_n_ species are indicative of the incorporation of a water molecule into the chain, leading to a structure that is likely formed by a linear-core (or star-like-core if the linear-core is short enough) and branches (referred above as *bl*PG).^26^ A qualitative analysis clearly evidence that an increase of *W*_n_ specimens occurs in samples prepared in the presence of water and that the higher the water amount, the higher the relative peak intensity of *W*_n_ compared to *C*_n_. The data also show that, even with a significant excess of water in a system exhibiting strong acidity ([H_2_O]_0_/[B(C_6_F_5_)_3_]_0_**=** 600/1),^42^*C*_n_ species are still formed, confirming that cyclic chains are the thermodynamically favored product. Moreover, these data also indicate that *bl*PG cannot be obtained with topological purity, that is, without the presence of *bc*PG units. For that reason, hereafter, we refer to samples obtained under wet conditions as a mixture of *bc*PG and *bl*PG structures (*bc+bl*PG).

**Figure 3:**
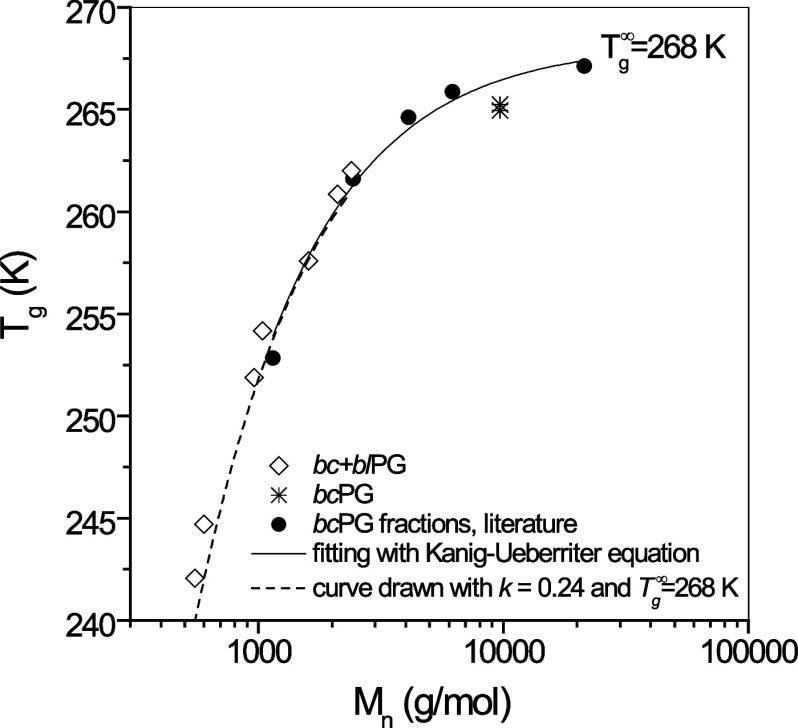
DSC *T*_g_ measured for *bc+bl*PG and *bc*PG synthesized in this study, and that obtained from literature for *bc*PG fractions.^21^ The solid line represents the fit using eq 5, while the dashed line corresponds to the curve described by eq 5 using the previously determined fit parameters.

The presence of *bl*PG chains in *bc*PG was confirmed not to affect a melt property such as *T*_g_ (Figures 3and S4). Plotting the molecular weight dependence of the *T*_g_ for both *bc+bl*PG and *bc*PG, along with previously reported data for *bc*PG fractions,^21^ revealed an identical trend. All data points converged onto a single curve described by the Kanig–Ueberreiter eq (eq 5),^43^ where the constant *k* was determined to be 0.24 g/molK and the high molecular weight limiting value, *T*_g_^*∞*^, was found to be 268 K. As reported by some of us, the presence of branches in PGs exerts a more significant influence on the *T*_g_ compared to the PG topology.^21^ This likely explains why the *T*_g_ was not sensitive to the presence of *bl*PG chains in the *bc+bl*PG mixture.
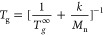
5

### Small-Angle X-Ray Scattering

To further analyze the structure and chain conformation of the synthesized PGs in the absence and presence of water, small-angle X-ray scattering (SAXS) experiments of aqueous solutions of PG samples were performed. SAXS data were obtained for PG fractions of different molecular weights generated by fractional precipitation from samples of similar DB. Five fractions were generated from Entries 7/F1–F5, and 6 fractions from Entry 8/F1–F6. Other nonfractionated samples were also measured (Entries 4, 5, and 9). We expect that fractionation of *bc+bl*PG (Entry 8) yields a mixture with a similar composition due to the comparable chemical nature of both components, *bl*PG and *bc*PG.

**Figure 4:**
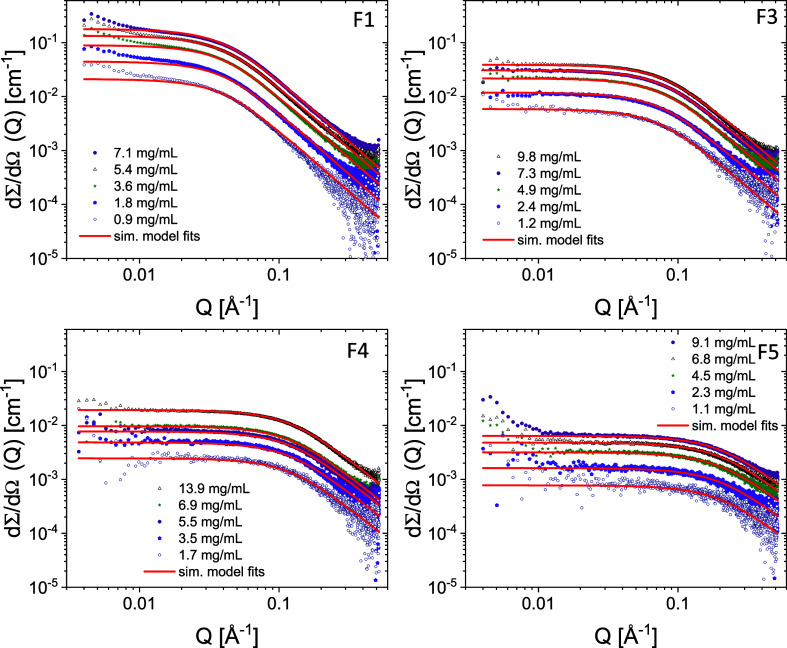
SAXS scattering profiles of aqueous solutions of representative fractions of Entry 7 (dry). The solid lines correspond to the model fits using eq 4.

**Figure 5:**
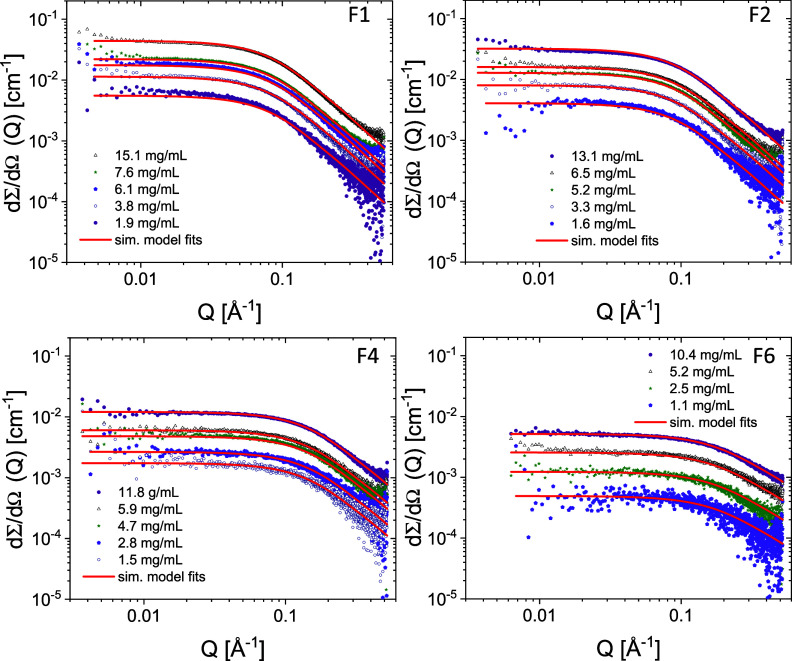
SAXS scattering profiles of aqueous solutions of representative fractions of Entry 8 (wet). The solid lines correspond to the model fits using eq 4.

The SAXS data, including the data fits, are shown for various samples at different concentrations in Figures 4 and 5. The scattering curves resemble the typical scattering patterns of polymers in solution, with a Guinier-like plateau at low *Q* in the double logarithmic representation, followed by a close to *Q*^–2^ behavior at high *Q*. The data thus show that the chains are molecularly dispersed without aggregation, except Entries 7/F1 and 7/F5, which show a slight upturn at low *Q* for the higher concentration, indicating larger clusters. In order to obtain quantitative information about the chain conformation (*R*_g_ and fractal dimension, *d*_f_) and extract the weight-average molecular weight (*M*_w_), we employed a form factor analysis described in the experimental section. The results are reported in Table 3. The *M*_w_ values found by SAXS are in agreement with those obtained by GPC-RI-MALS.

**Table 3:** Molecular Masses and Fitting Data of SAXS Curves for *bc*PG Fractions (Entry 7), *bc+bl*PG Fractions (Entry 8) and Nonfractionated *bc+bl*PG Samples (Entries 4, 5, and 9)

		GPC^a^		SAXS^b^		
samples		*M*_w_ (kg/mol)	Đ	*M*_w_ (kg/mol)	*R*_g_ (Å)	*d*_f_
entry 7	F1	38.8	1.3	39.0	37 ± 2	2.2
	F2	14.6	1.4	16.1	27 ± 1	2.2
	F3	7.0	1.5	12.2 ± 0.4	20 ±1	2.2
	F4	2.9	1.4	3.2 ± 0.2	12 ±1	2.1
	F5	1.0	1.3	1.5 ± 0.1	8 ±1	2.0
entry 8	F1	6.8	1.2	6.6 ±0.5	18 ±2	2.1
	F2	4.9	1.4	5.6 ±0.3	16 ±1	2.1
	F3	4.5	1.1	4.2 ± 0.3	14 ± 1	2.1
	F4	3.4	1.2	3.4 ± 0.2	12 ± 1	2.1
	F5	3.4	1.3	2.2 ± 0.2	11 ± 1	2.1
	F6	1.5	1.1	1.1 ± 0.1	8 ± 1	1.7
entry 4	-	4.9	2.6	5.9 ± 0.5	17 ± 2	2.0
entry 5	-	1.9	1.2	1.1 ± 0.1	7 ± 1	1.7
entry 9	-	1.0	1.2	1.0 ± 0.2	8 ± 1	1.7

a Obtained by GPC with RI-MALS detection.

b Determined by fitting and analysis of SAXS curves.

**Figure 6:**
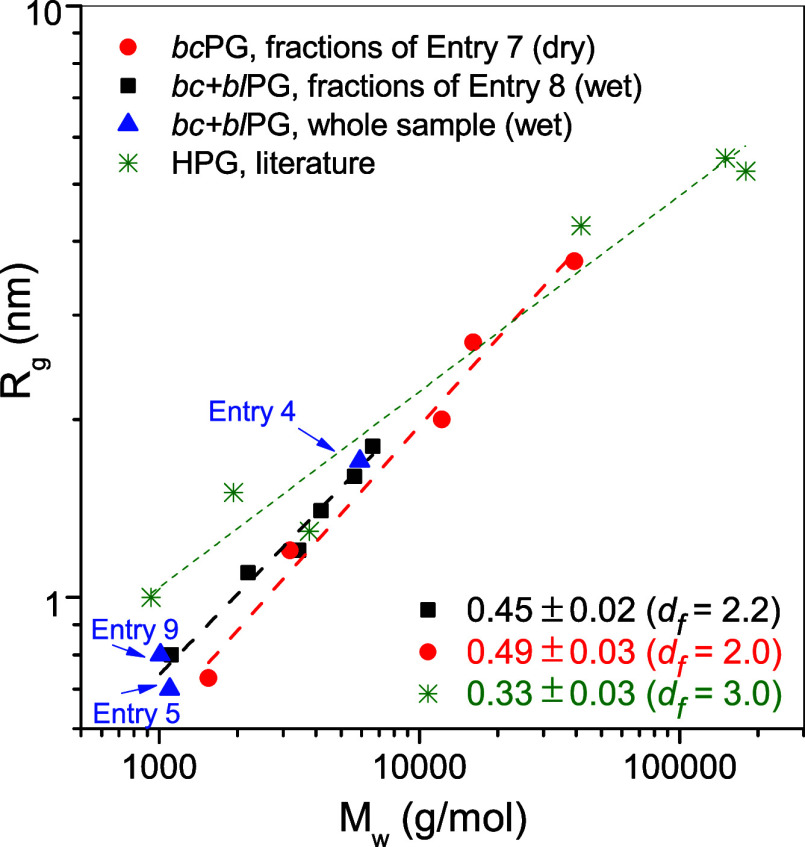
Log–log plot of *R*_g_ vs *M*_w_ obtained by SAXS, indicating slope and fractal dimension (*d*_f_) values. Literature data of HPG obtained by SANS.^44^

The scaling behavior of *R*_g_ versus *M*_w_ extracted from the fits is depicted in Figure 6, together with the data collected from the literature for the hyperbranched polyglycerol (HPG).^44^ The results show similar molecular weight dependence of *R*_g_ for PG samples obtained in the absence and presence of water but different *R*_g_ values for similar molecular weight. From the slopes, *d*_f_ = 2.2 was obtained for samples obtained in the presence of water and *d*_f_ = 2.0 for those obtained in dry conditions. These values are in agreement with those of randomly branched polymers (*d*_f_ = 2 in a good solvent, *d*_f_ = 2.28 in a θ solvent).^45,46^ In contrast, HPG shows a *d*_f_ = 3 corresponding to a globular structure.^44^ Interestingly, the direct form factor analysis indicated a slope at high *Q* that corresponds to *d*_f_ values for several polymers about 2.2 (Table 3, Entry 7/F1–F3). This suggests that the polymers are locally more compact, likely due to the branching. The samples exhibiting *d*_f_ values about 1.7–1.8 are the fractions of the lowest *M*_w_ (Entry 7/F5 and Entry 8/F6) and the nonfractionated samples (Entries 4, 5, and 9). These low *d*_f_ values may point to a low degree of branching in agreement with the low DB reported in Table 2 for Entries 4, 5, and 9, and the smaller DB found for fractions of lower *M*_w_.^21^ Moreover, the *R*_g_ data of nonfractionated *bc+bl*PG samples lie on the fitted line for fractionated *bc+bl*PG samples of Entry 8, which show that their *R*_g_ values do not specifically show differences that can be attributed to a different topological composition related to their synthesis with different amounts of water.

The data also show smaller *R*_g_ values by a factor of 1.14 for PGs obtained under dry conditions compared to those obtained in the presence of water. The cyclic core of *bc*PG generated in the absence of water could be responsible for a more compact structure despite the branching. Atomistic molecular dynamic simulations of cyclic and linear poly(vinyl alcohol)-*graft*-poly(ethylene oxide) bottlebrush polymers (BBPs) in aqueous solution showed that the cyclic-core structure is, in general, more compact than the linear analogue.^47^ Moreover, they found that the shape of the cyclic-core BBPs changes from donutlike to disklike to starlike with increasing side chain length and that the shape of the linear-core BBPs changes from an expanded coil to a rod/cylinder. Our PGs, despite having a less defined side chain structure due to branching, bear a resemblance to those of BBPs, where the cyclic and linear cores dominate their size. Another analogy of our experimental data with that of atomistic simulations is the *R*_g(wet)_/*R*_g(dry)_ = 1.14 found in our experiments for identical *M*_w_ compared to *R*_g(L)_/*R*_g(C)_ = 1.20 found at identical side chain length in the simulations.^47^

### Cytotoxicity and Cellular Uptake of PGs

**Figure 7:**
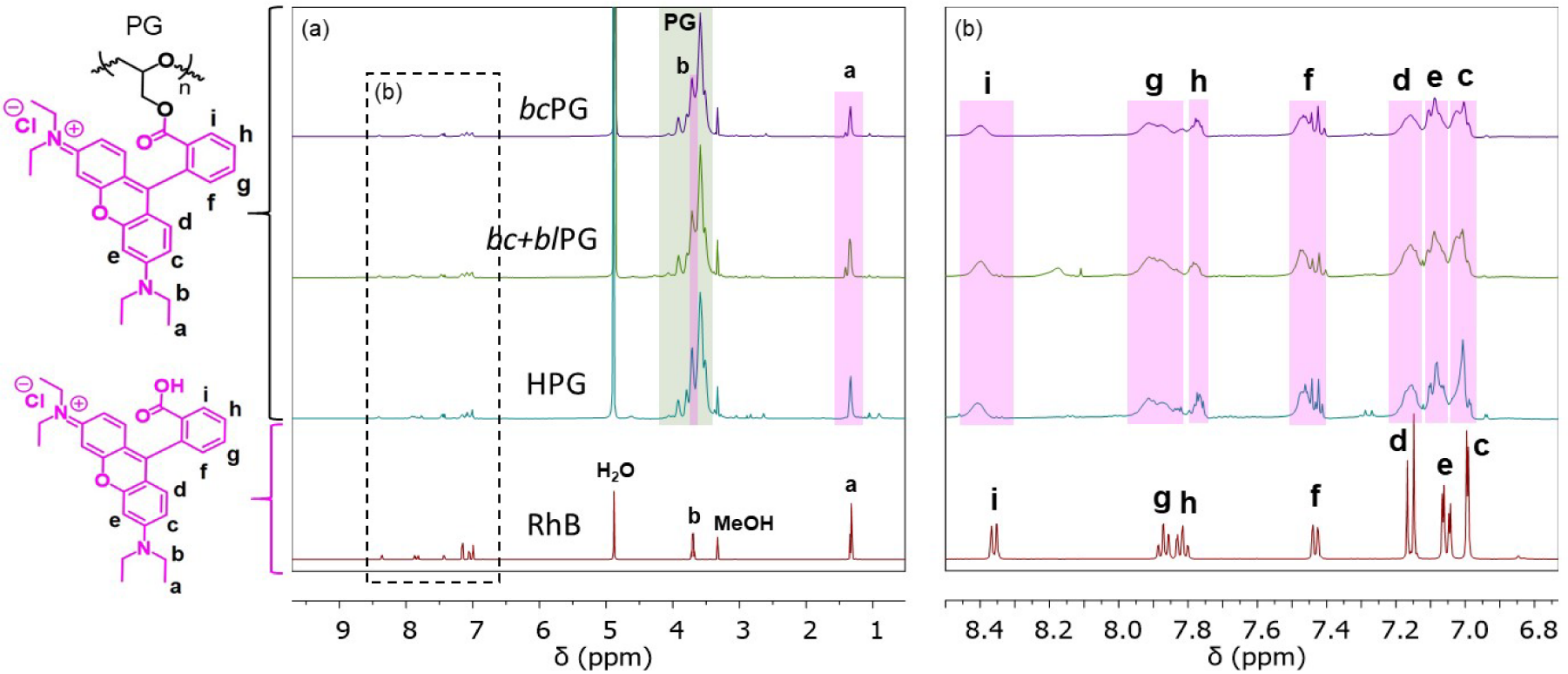
(a) ^1^H NMR spectra in methanol-*d*_4_ of RhB and RhB-functionalized PG: *bc*PG, *bc+bl*PG, and HPG. (b) Inset: Assignment was performed according to ref (49).

**Figure 8:**
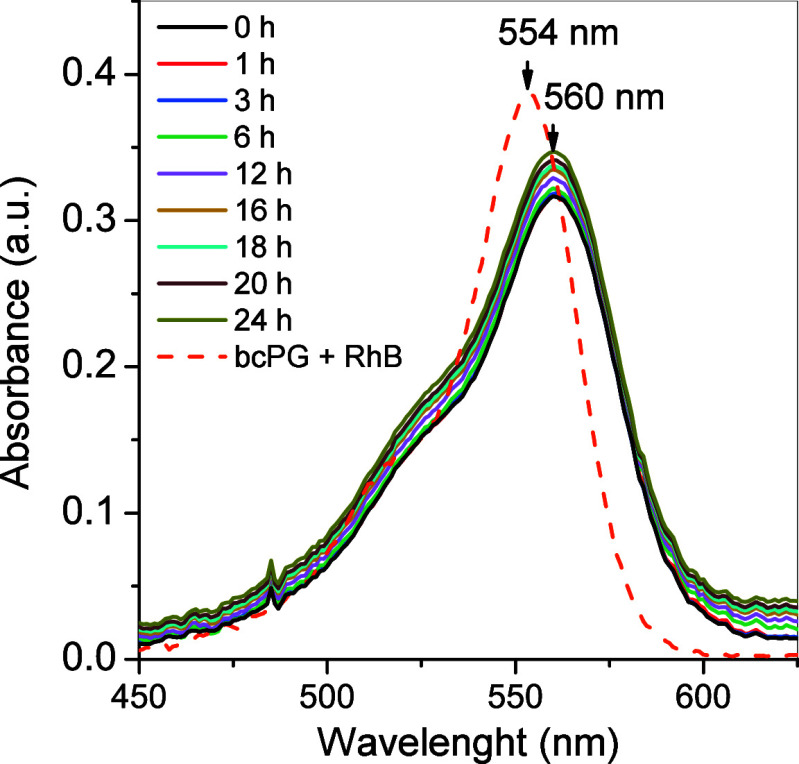
Monitoring of UV–vis absorption of RhB-*bc*PG as a function of time in physiological conditions (PBS pH 7.4, 37 °C). Reference sample: a mixture of *bc*PG and 3 mol % of RhB.

To evaluate the effect of the PG structure on their cytotoxicity, representative PG samples of similar molecular weight and degree of branching, obtained in “dry” and “wet” conditions were analyzed (Table 4). As a reference sample, a globular HPG sample was synthesized following a standard protocol.^34^ To generate fluorescently labeled polymer chains, PGs were functionalized with rhodamine B (RhB) *via* esterification of PG hydroxyls with RhB carboxylic acid. ^1^H and^13^C NMR, FTIR, UV–vis, and GPC characterization confirmed the RhB grafting. ^1^H NMR spectra of RhB-functionalized PG (RhB-PG) exhibited signals corresponding to RhB moieties significantly broadened in the aromatic region (Figure 7). Quantification of the amounts of RhB per hydroxyl group of PG by ^1^H NMR resulted in about 3 mol %. The formation of ester bonds was confirmed by ^13^C NMR and FTIR through a peak shift of the RhB C=O signal from 166.7 to 165.2 ppm after esterification (Figure S5), and a peak shift of the RhB C=O stretching band from 1706 to 1716 cm^–1^ (Figure S6). UV–vis detection in GPC experiments revealed a transition in the polymer absorbance from nonabsorbance to absorbance at 554 nm after functionalization (Figure S7). The UV–vis absorption band of RhB-grafted polymers exhibited a red shift in peak maxima from 554 (physical mixture of RhB and PG) to 560 nm in phosphate-buffered saline (PBS) of pH 7.4 at 37 °C upon functionalization (Figure 8) in agreement with literature data.^48^ The position of this band remained unchanged for 24 h, indicating that RhB remained covalently attached to PGs for at least 24 h.

**Table 4:** Molecular Characteristics of RhB-Functionalized PG Samples

sample	*M*_n_(kg/mol)	Đ	DB	RhB(mol %)
*bc*PG^a^	4.8	1.5	0.45	2.6
*bc+bl*PG^b^	5.5	1.2	0.42	3.3
HPG	5.0	1.6	0.50	3.3

a Entry 7/F3.

b Entry 8/F1.

**Figure 9:**
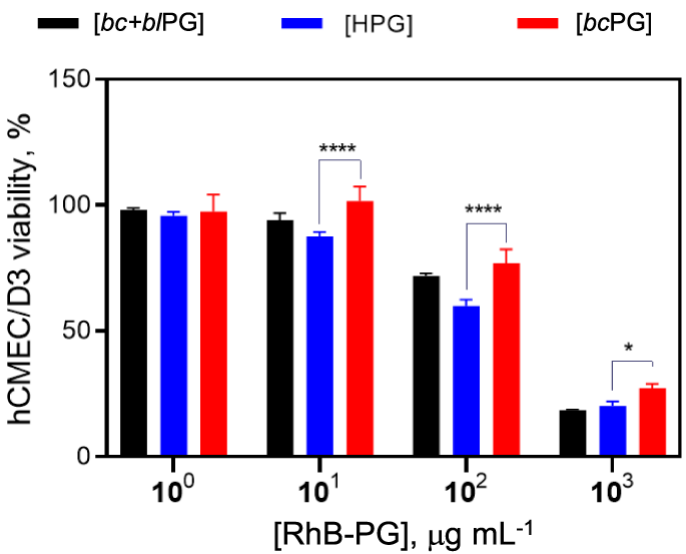
*In vitro* cell viability of RhB-PG polymers at varying concentrations in endothelial hCMEC/D3 cells. Cells were treated for 24 h, and then the cell viability was assessed by MTT assay. Results are presented as the mean of three independent experiments ± SD considering untreated cells as 100% of cell viability. Statistical analysis was carried out by t-test. *p < 0.05; **** *p* < 0.0001.

Then, hCMEC/D3 cells (used as a cellular model) were treated with different concentrations of RhB-PG for 24 h, and cell viability was measured by MTT assay.^50^ The results showed that RhB-PG doses of up to 100 μg mL^–1^ were well-tolerated by the tested endothelial cell line, as evidenced by cell viability exceeding 50% (Figure 9). A substantial reduction in cell viability was observed at a RhB-PG dose of 1000 μg mL^–1^. Overall, RhB-bcPG showed a less cytotoxic effect in comparison to the other PG structures. These results contrast with previous studies on LPG and HPG of low (∼6 kDa)^14^ and high (∼100 kDa)^13^ molecular weights, where no effect of the polymer topology on the cytotoxicity was found using human umbilical vein endothelial cells (HUVECs) at polymer concentrations up to 10 mg mL^–1^.

### *In Vitro* Endothelial Permeability

**Figure 10:**
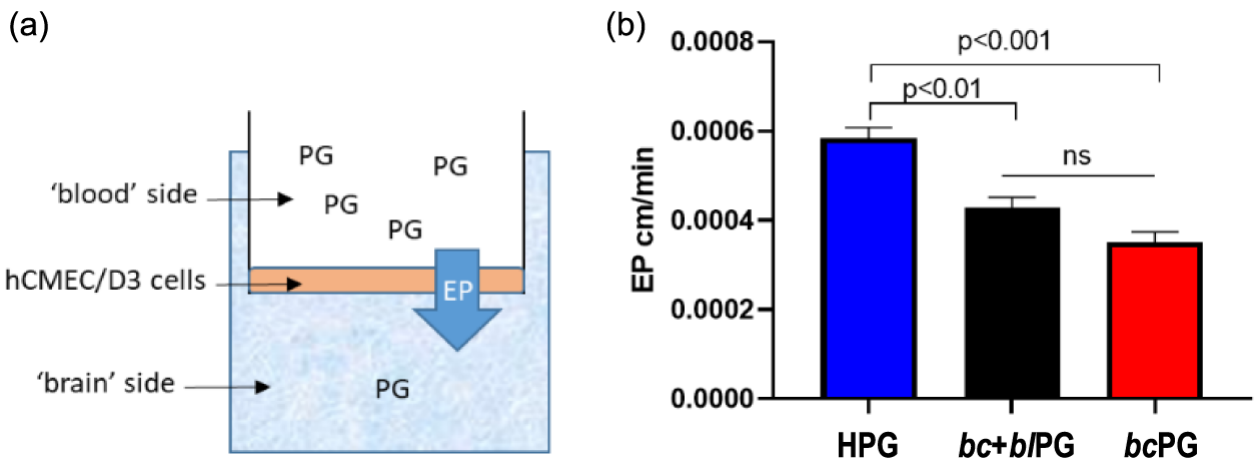
Endothelial permeability (EP) of PGs in a transwell model of the blood–brain barrier. (a) Graphical representation of the transwell system used to mimic the BBB. (b) Endothelial permeability of RhB-PGs (different topology) calculated by measuring the fluorescence in the “brain” side compartment over time (up to 3 h, at 37 °C). 10 μg/mL of RhB-PG was added to the “blood” compartment. Data are expressed as the mean ± SD of at least three independent measurements, each of which is in triplicate.

The influence of the topology of branched PGs on their ability to cross the BBB was assessed by using an *in vitro* transwell model made by hCMED/D3 (Figure 10a). This is the most utilized and known *in vitro* model to evaluate the ability of drugs, molecules, polymers, and nanoparticles to overcome a cell monolayer made by brain capillary endothelial cells.^35^ The results showed that the highest endothelial permeability (EP) was detected for RhB-HPG, in comparison to the other PGs tested (Figure 10b), suggesting that the topology of the polymers can affect the ability to cross a cell monolayer. We hypothesize that their different shapes and/or sizes in a water-enriched environment may affect their interaction with cell membranes and subsequently the EP. HPG exhibits a globular structure, unlike *bc*PG and *bc+bl*PG, and *bc*PG is more compact (smaller *R*_g_) than *bc+bl*PG. Considering that size and shape are critical physical factors affecting BBB crossing,^51^ this aspect will be subjected to further investigation. The EP of free RhB was 2.91 × 10^–2^ cm/min, which was 100-fold higher than that of RhB-PGs. This result confirms that RhB was stably bound to PG, and the measured EP reflects the permeability of RhB-PGs.

**Figure 11:**
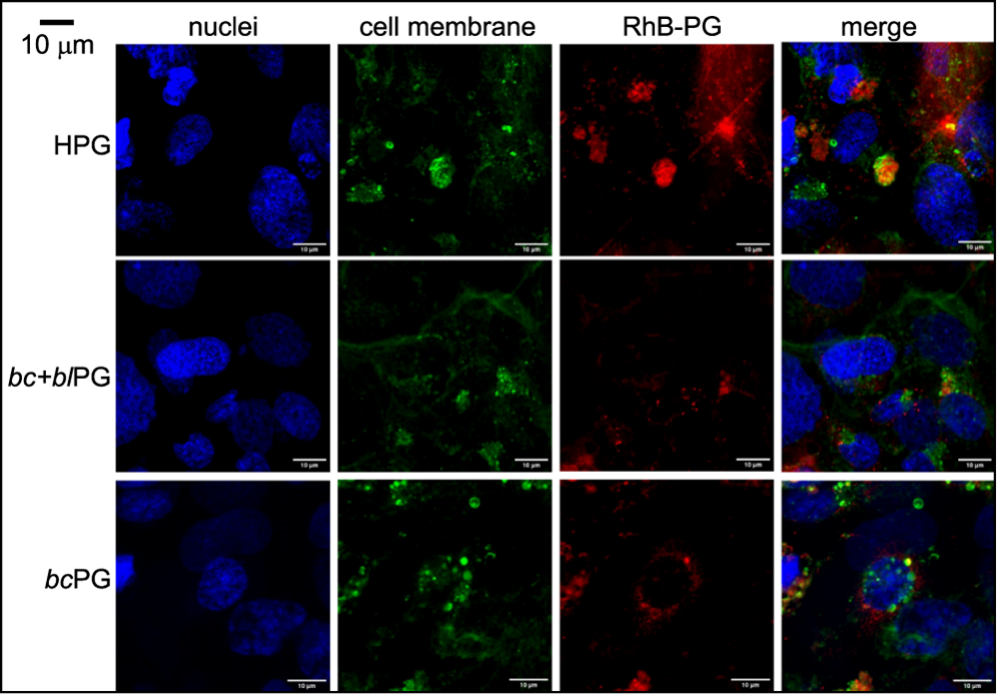
Confocal images showing the RhB-PG passage through the transwell model made by hCMED/D3. All RhB-PGs are inside the cell monolayer showing that BBB crossing occurs though the cells. RhB-HPG exhibits a higher cellular uptake compared to other PG structures. RhB-*bc*PG mainly localizes around the cell nuclei.

Finally, to check if the RhB-PG passage was between cell spaces or across the cells, confocal microscopy imaging was performed on hCMEC/D3 cells. The results (Figure 11) showed that all RhB-PGs were detected inside the cells, suggesting that BBB crossing occurred through the cells. A higher cellular uptake for RhB-HPG compared with the other PG structures was noticed, in agreement with the highest EP value found for RhB-HPG. Again, the PG conformation in a water-enriched environment may affect the interaction of the polymers with the cell membrane. Interestingly, RhB-*bc*PG was mainly localized around cell nuclei, unlike RhB-HPG which was not. RhB-*bc+bl*PG also seems localized around the nuclei, although its detection was less clear. The results confirm the ability of PG structures to successfully cross the BBB, as those found for polymeric micellar nanocarriers of about 30 nm of diameter.^52^ Considering that our PG structures do not form micelles and that their diameters in water are only 3–4 nm (see *R*_g_ values in Figure 6), it is likely that the micrometer-size fluorescent spots detected in Figure 11 are due to their aggregation and accumulation within specific parts of the cell. Comparing the EP with functionalized nanoliposomes to cross the BBB (EP in the order of 10^–5^ cm/min),^35^ RhB-PGs showed higher values (EP in the order of 10^–4^ cm/min). While *in vivo* rodents are typically used to evaluate the biocompatibility, biodistribution, and potential toxicity of polymers, *in vitro* models are commonly used to predict the behavior of macromolecules, drugs, and polymers.^53^ PGs have previously been shown to exhibit minimal polymer accumulation in vital organs after intravenous injection compared to other polymers.^3^ In addition, they have been well tolerated in mice and rats with no evidence of immunogenicity reported to date. Our findings with an *in vitro* model open the possibility of using current PGs structures as brain delivery systems.

## Conclusions

Under dry conditions, *bc*PG is obtained with topological purity. As the water content increases in the polymerization of glycidol with B(C_6_F_5_)_3_, there is a corresponding increase in the formation of *bl*PG structures. The formation of cyclic species persisted even in the presence of a significant excess of water, confirming that cyclic chains are the thermodynamically favored product. Therefore, achieving topological purity for *bl*PG was not feasible and only mixtures of *bc*PG and *bl*PG (*bc+bl*PG) structures could be obtained. This had important implications for the final properties of the products obtained under dry or wet conditions.

The structure and conformation of the PGs in solution were characterized by SAXS. A power-law scaling relationship between the radius of gyration and molar mass was obtained, yielding a fractal dimension of the order of two for PGs obtained under both dry and wet conditions, consistent with the formation of randomly branched structures and in contrast to the globular and compact nature of HPG. In addition, an R_g(wet)_/R_g(dry)_ ratio of 1.14 was obtained, indicating that the pure *bc*PG formed under dry conditions is more compact than the mixture of *bc*PG and *bl*PG structures obtained in the presence of water.

The differences in polymer shape or size may be the factor responsible for the different biological responses between HPG and *bc*PG. HPG showed higher endothelial permeability and cellular uptake compared with *bc*PG and *bc+bl*PG. *bc*PG (and apparently *bc+bl*PG) was localized around the cell nuclei, whereas HPG did not exhibit this behavior. Overall, *bc*PG showed a less cytotoxic effect compared to the other PG structures.

Our study demonstrates the importance of finely controlling the polymerization conditions of glycidol with B(C_6_F_5_)_3_ to minimize the unwanted presence of water, which can produce *bl*PG structures that affect the biological activity of the topological analogue *bc*PG. Our results also suggest potential applications of PG structures in brain delivery systems and highlight the importance of polymer topology in the BBB crossing *in vitro*.

## Supporting Information Available

Comparison of degree of branching (DB) values obtained from two different equations used in hyperbranched polymers (Table S1); DEPT-135 and inverse gated ^13^C NMR (D_2_O) spectra of Entry 4 (Figure S1); ^1^H–^13^C (DEPT-135) HSQC (D_2_O) spectrum of Entry 4 (Figure S2); Inverse gated ^13^C NMR (D_2_O) spectra of Entries 1, 3, and 7 (Figure S3); DSC data of representative samples (Figure S4); ^13^C NMR spectra (in methanol-*d*4) of RhB and RhB-functionalized PG: *bc*PG, *bc+bl*PG and HPG (Figure S5); FTIR spectra of RhB-functionalized PGs, RhB, and *bc*PG (Figure S6); GPC data in DMF + 0.1% LiBr of RhB-functionalized PG (Figure S6)(PDF)
