# Supplementary material for: In Vitro Biocompatibility and Endothelial Permeability of Branched Polyglycidols Generated by Ring-Opening Polymerization of Glycidol with B(C6F5)3 under Dry and Wet Conditions
Source: Biomacromolecules. 2024 May 4;25(6):3583–95. doi: 10.1021/acs.biomac.4c00210 (PMC11170947; doi:10.1021/acs.biomac.4c00210)
Supplement: Supplementary file 1 — bm4c00210_si_001.pdf [file bm4c00210_si_001.pdf]

Supporting Information

In Vitro Biocompatibility and Endothelial Permeability of Branched Polyglycidols Generated by Ring-Opening Polymerization of Glycidol with $B(C_6F_5)_3$ under Dry and Wet Conditions

Carlo Andrea Pagnacco,[a,b] Marcelo H. Kravicz,[c] Francesco Saverio Sica,[c] Veronica Fontanini,[c,d] Estibaliz González de San Román,[e] Reidar Lund,[*f,g] Francesca Re,[*c] Fabienne Barroso-Bujans[*a,b,h]

[a]Donostia International Physics Center (DIPC), Paseo Manuel Lardizábal 4, Donostia−San Sebastián, 20018, Spain

[b]Centro de Física de Materiales, CSIC-UPV/EHU, Paseo Manuel Lardizábal 5, Donostia−San Sebastián, 20018, Spain

[c]School of Medicine and Surgery, University of Milano-Bicocca, Milano, 20854, Italy

[d]Department of Life Sciences, University of Trieste, Trieste, 34127, Italy

[e]POLYMAT, University of the Basque Country UPV/EHU, Joxe Mari Korta Center, Avda. Tolosa 72, Donostia−San Sebastián, 20018, Spain

[f]Department of Chemistry, University of Oslo, Postboks 1033, Blindern, Oslo, 0315, Norway

[g]Hylleraas Centre for Quantum Molecular Sciences, University of Oslo, Postboks 1033, Blindern, Oslo, 0315, Norway

[h]IKERBASQUE - Basque Foundation for Science, Plaza Euskadi 5, Bilbao, 48009, Spain

Supporting Information

Supporting tables

Table S1. Comparison of degree of branching (DB) values obtained from two different equations used in hyperbranched polymers.

Entry	M_n (GPC) (kg/mol)	DB = 2D/(2D+L)	DB = (D+T)/(D+T+L)
1	9.7	0.46	0.46
2	3.5	0.45	0.42
3	1.9	0.43	0.47*
4	1.9	0.38	0.48*
5	1.6	0.30	0.48*
6	0.6	0.39	0.44*
7	9.7	0.46	0.45
8	3.8	0.43	0.45
9	0.8	0.27	0.47*
10	0.3	0.20	0.53*

*Overestimated DB values in samples with low molecular weight or low degree of branching, in agreement with reference S1. $L = L_{1,3} + L_{1,4}$ and $T = T_1 + T_2$.

Supporting figures

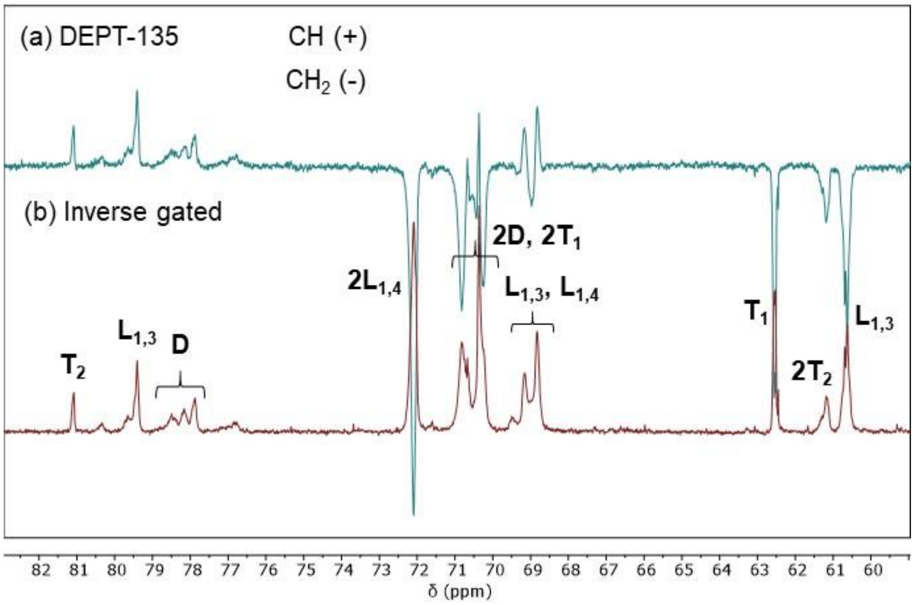

Figure S1. (a) DEPT-135 and (b) inverse gated ^{13}C NMR (D_2O) spectra of Entry 4.

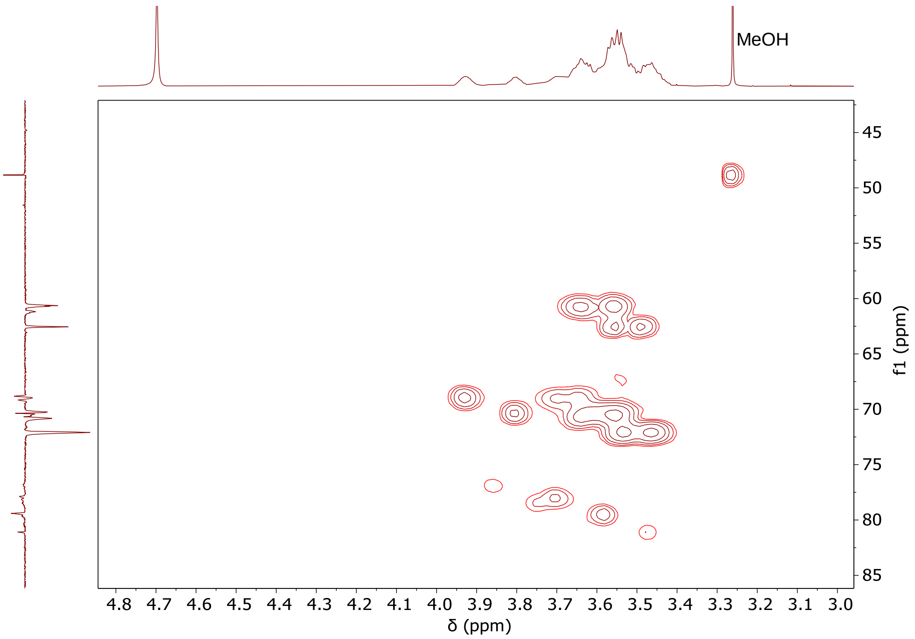

Figure S2. ^{1}H - ^{13}C (DEPT-135) HSQC (D_2O) spectrum of Entry 4.

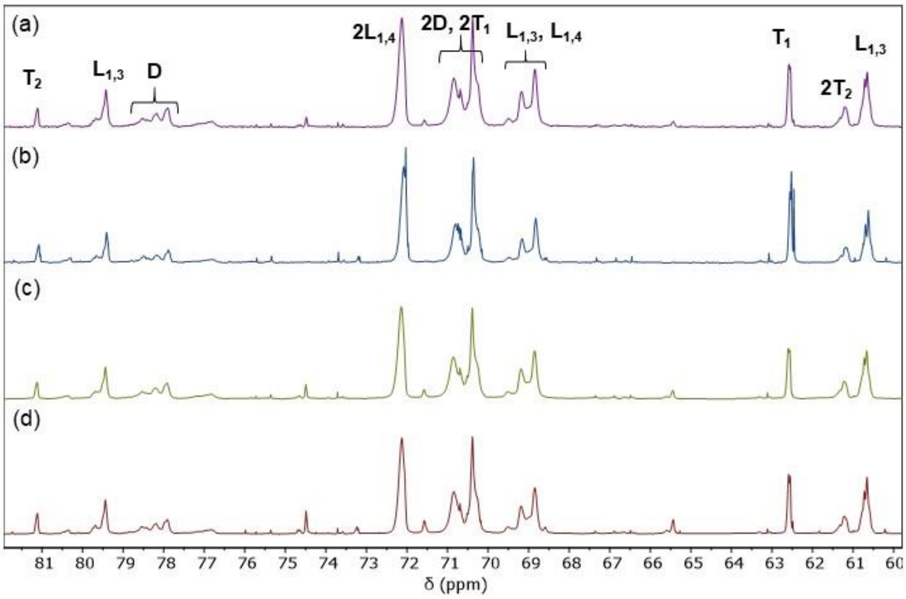

Figure S3. (a) Inverse gated ^{13}C NMR (D_2O) spectra of Entries (a) 1, (b) 3, (c) 7 and (d) 8.

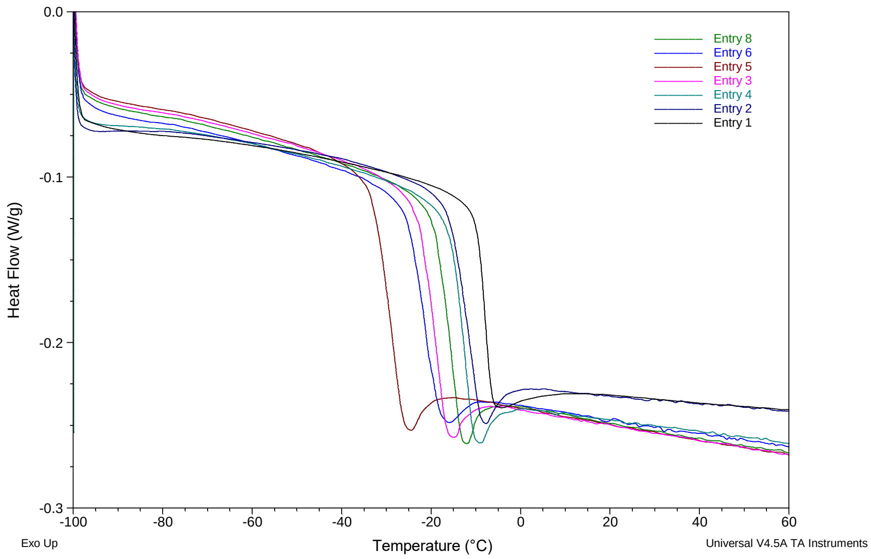

Figure S4. DSC data of representative samples (Entries of Table 1) obtained at a heating rate of 10 ºC/min (2nd heating run).

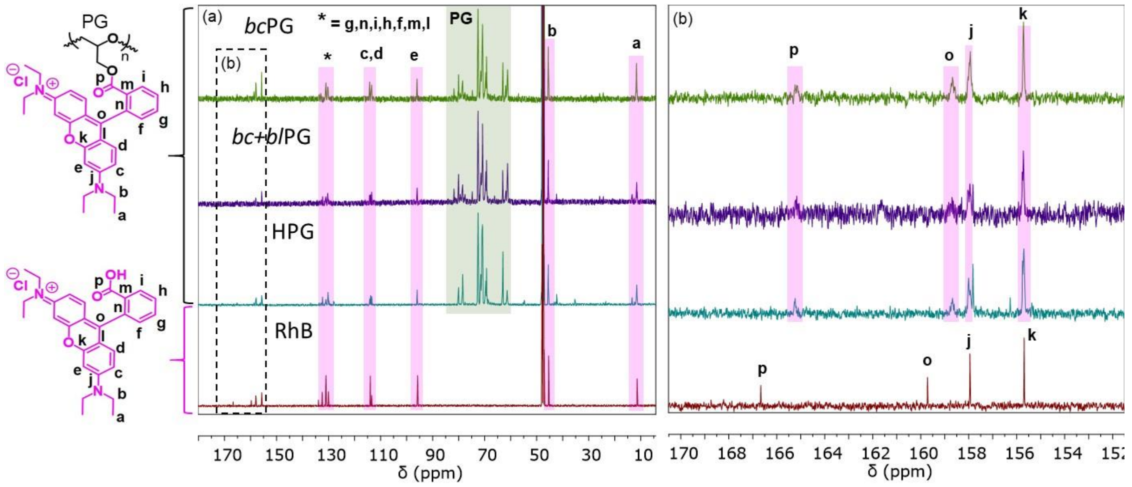

Figure S5. (a) ^{13}C NMR spectra (in methanol-d_4) of RhB and RhB-functionalized PG: *bc*PG, *bc*+*bl*PG and HPG. (b) Inset. Assignment was performed according to reference S[2].

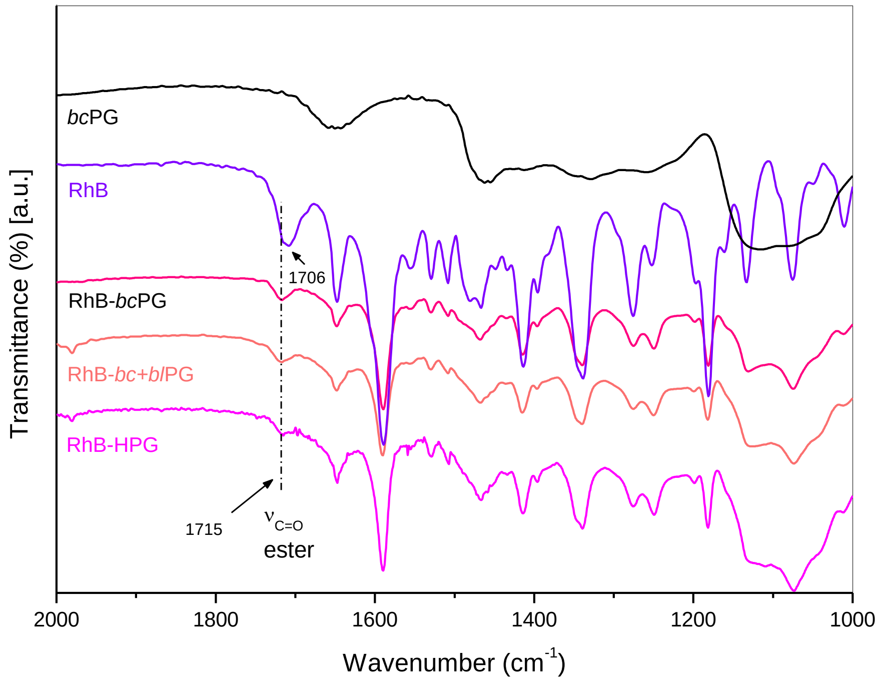

Figure S6. FTIR spectra of RhB-functionalized PGs, RhB and bcPG in the spectral range of 1000-2000 cm^{-1}.

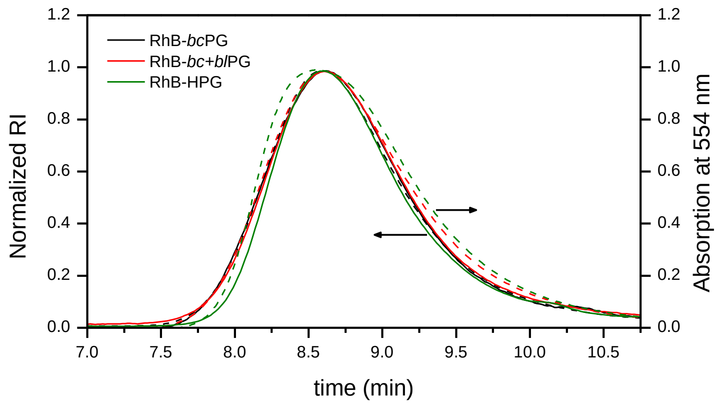

Figure S7. GPC data in DMF + 0.1% LiBr of RhB-functionalized PG registered with refractive index (RI) and UV-Vis (at 554 nm) detectors.

References

(1) Hölter, D.; Burgath, A.; Frey, H., Degree of branching in hyperbranched polymers. *Acta Polym.* **1997,** *48* (1-2), 30-35.

(2) Ding, L.; Hayakawa, T.; Kakimoto, M.-a., Synthesis and Characterization of Hyperbranched Poly(siloxysilane) Possessing Rhodamine B as Terminal Group. *Polym. J.* **2007,** *39* (6), 551-557.
